# Real-world data on the use of subcutaneous daratumumab plus bortezomib, thalidomide, and dexamethasone in transplant-eligible patients with newly diagnosed multiple myeloma

**DOI:** 10.1007/s00277-025-06365-3

**Published:** 2025-04-30

**Authors:** Vania Hungria, Fernanda Lemos Moura, Abel Costa, Eduardo Flávio Oliveira Ribeiro, Paulo Soares, Juliana Souza Lima, Lisa Aquaroni Ricci, Celso Arrais-Rodrigues, Fabio Moore Nucci, Marinus de Moraes Lima, Roberto Jose Pessoa de Magalhães Filho, Amitabha Bhaumik, Trilok Parekh, Fredrik Borgsten, Robin Carson, Damila C. Trufelli, Edvan de Queiroz Crusoe

**Affiliations:** 1 Clinica São Germano, Rua Comendador Miguel Calfat 165, São Paulo, SP 04537-080 Brazil; 2 Fundação Antonio Prudente - AC Camargo Cancer Center, São Paulo, SP Brazil; 3 Instituto D’Or de Pesquisa e Ensino, São Paulo, SP Brazil; 4 Hospital Santa Lúcia, Brasilia, Distrito Federal, Brazil; 5 Instituto D’Or de Pesquisa e Ensino, Brasilia, Distrito Federal, PE Brazil; 6 Instituto de Hematologia e Oncologia Curitiba, Curitiba, PR Brazil; 7 Instituto de Oncologia de Sorocaba, Sorocaba, SP Brazil; 8 Hospital Nove de Julho (DASA), São Paulo, SP Brazil; 9 Instituto de Pesquisa e Ensino, Rio de Janeiro, RJ Brazil; 10 Instituto D’Or de Pesquisa e Ensino, Recife, PE Brazil; 11 Complexo Hospitalar de Niterói, Niterói, RJ Brazil; 12 Johnson & Johnson, Titusville, NJ USA; 13 Johnson & Johnson, Raritan, NJ USA; 14 Johnson & Johnson, Spring House, PA USA; 15 Johnson & Johnson, São Paulo, SP Brazil; 16 Instituto D’Or de Pesquisa e Ensino, Hospital São Rafael, Salvador, BA Brazil

**Keywords:** Subcutaneous, Daratumumab, Multiple myeloma, Autologous stem cell transplant, Real-world

## Abstract

Subcutaneous daratumumab in combination with bortezomib, thalidomide, and dexamethasone (D-VTd), is approved for transplant-eligible patients with newly diagnosed multiple myeloma (NDMM). However, additional real-world data are needed to assess the effectiveness and safety of D-VTd in routine clinical practice. We conducted a noninterventional, multicenter, observational study across hematology centers in Brazil to gather real-world data on D-VTd in patients with NDMM who were eligible for autologous stem cell transplant (ASCT). Eligible patients must have completed 1 or more cycle of D-VTd, on or before September 30, 2022, to be included. Data were collected retrospectively from the start of D-VTd to the study inclusion visit using patient medical records and prospectively thereafter using electronic case report forms. As of the data cutoff (August 8, 2023), 49 patients were included. By the end of consolidation, 91.7% of patients achieved an overall response (partial response or better) and 89.6% achieved very good partial response or better. Forty-five (91.8%) patients underwent stem cell mobilization with a median stem cell yield of 5.7 × 10^6^ CD34+ cells/kg, and 44 (89.8%) patients underwent ASCT, among whom 43 out of 44 (97.7%) successfully completed ASCT. D-VTd was well tolerated, with a safety profile consistent with that previously known for daratumumab and VTd. Grade 3/4 neutropenia/febrile neutropenia and infections were reported in 32.7% and 18.4% of patients, respectively. Overall, results were consistent with the established profile of D-VTd, and these real-world effectiveness and safety results support the frontline use of subcutaneous daratumumab plus VTd in transplant-eligible patients with NDMM.

**Supplementary Information:**

The online version contains supplementary material available at 10.1007/s00277-025-06365-3.

## Introduction

Daratumumab is a human IgGκ monoclonal antibody targeting CD38 with a direct on-tumor [1, 2, 3, 4] and immunomodulatory [5, 6, 7] mechanism of action, demonstrating greater cytotoxicity toward multiple myeloma (MM) cells ex vivo compared with analogs of other CD38 antibodies [8]. With favorable results from several pivotal clinical studies, daratumumab is approved in a number of countries as a monotherapy for patients with relapsed or refractory MM, or in combination with standard-of-care therapies for patients with relapsed or refractory MM or newly diagnosed MM (NDMM) [9, 10, 11, 12, 13, 14, 15].

Notably, daratumumab has significantly improved clinical outcomes when included in frontline triplet [11] and quadruplet [13, 15, 16] regimens. In transplant-eligible patients with NDMM, the phase 3 PERSEUS study demonstrated that the quadruplet combination of subcutaneous (SC) daratumumab plus bortezomib, lenalidomide, and dexamethasone (D-VRd) significantly improved progression-free survival (PFS; hazard ratio [HR], 0.42 [95% confidence interval (CI), 0.30–0.59]; *P* < 0.001) and deepened clinical response (minimal residual disease [MRD] negativity at 10^–5^ sensitivity, 75.2% vs. 47.5%; *P* < 0.001) versus VRd alone at a median follow-up of 47.5 months [16]. Furthermore, in the phase 3 CASSIOPEIA study, the quadruplet combination of intravenous (IV) daratumumab plus bortezomib, thalidomide, and dexamethasone (D-VTd) significantly improved PFS (HR, 0.61 [95% CI, 0.52–0.72]; *P* < 0.0001) and deepened clinical response (MRD negativity at 10^–5^ sensitivity, 33.7% vs. 20.3%; *P* < 0.0001) versus VTd alone at a median follow-up of 80.1 months in transplant-eligible patients with NDMM [13]. Together, these studies underscore the use of daratumumab as a pivotal component in the treatment landscape of NDMM.

Despite its favorable clinical benefits, daratumumab IV is commonly associated with infusion/injection-related reactions [17], which can lead to longer infusion times, or “chair time,” for patients. The SC formulation of daratumumab (daratumumab 1,800 mg co-formulated with recombinant human hyaluronidase PH20 [2,000 U/mL; ENHANZE^®^ drug delivery technology, Halozyme, Inc.]) [18] was developed to reduce administration time, infusion/injection-related reactions, and overall treatment burden [19]. In the phase 3 COLUMBA study, daratumumab SC was noninferior to daratumumab IV as monotherapy, with comparable efficacy, pharmacokinetics, and safety [20]. Following these findings, daratumumab SC was approved in the United States [18], Europe [21], and Brazil [22] as a monotherapy and in combination with other therapies. The pivotal phase 2 PLEIADES study explored the safety and efficacy of daratumumab SC with standard-of-care regimens, including the proteasome inhibitor/immunomodulatory drug/steroid combination bortezomib, lenalidomide, and dexamethasone (VRd) in transplant-eligible patients with NDMM, with the primary endpoint of very good partial response or better [≥ VGPR] met and no new safety concerns identified [23].

While clinical trials are vital in the evaluation of new therapies and combinations, their controlled protocols often do not reflect the diversity and complexities of routine clinical practice [24]. Real-world studies address these gaps by providing insights into routine clinical practice and the outcomes and challenges experienced by patients, which can better direct treatment decision making [24]. Daratumumab IV plus VTd has been clinically supported [25], daratumumab SC is noninferior to IV [20], and daratumumab SC is safe and effective in combination with a proteasome inhibitor and immunomodulatory drug [23]; however, there is still a need for real-world evidence on the safety and effectiveness of daratumumab SC plus VTd from routine clinical practice.

Here we present findings from a noninterventional, multicenter study assessing the safety and clinical outcomes of daratumumab SC plus VTd (hereafter denoted as D-VTd) in patients with NDMM who are eligible for autologous stem cell therapy (ASCT) within a real-world clinical practice context in Brazil.

## Methods

### Study design and patients

This noninterventional, multicenter, observational study was conducted as a Post-Authorization Safety Study to meet a specific request from the US Food and Drug Administration, with the primary objective of evaluating the safety and clinical outcomes of D-VTd induction/consolidation treatment. The study enrolled treatment-naïve patients with NDMM who were considered candidates for ASCT at the start of D-VTd induction/consolidation treatment from multiple specialized and community private centers with a specialty in hematology in Brazil. In addition to their MM diagnosis, patients had to be eligible for D-VTd treatment according to local practice and must have completed ≥ 1 cycle of D-VTd treatment on or before September 30, 2022. Patients with contraindications for D-VTd treatment, who had received an investigational drug (including investigational vaccine) or used an investigational medical device within 60 days prior to study initiation, who were currently enrolled in another interventional study, or who were already receiving D-VTd and had current plans to or were foreseeably planning to replace any component of D-VTd with another treatment, were excluded from the study. However, patients were not prohibited from changing their treatment regimen later during study participation. The decision to treat with D-VTd must have been made prior to and independent of the patient’s inclusion in the study to ensure the study did not influence or alter the standard of care they were receiving. To minimize bias, consecutive eligible patients were enrolled in the study.

All aspects of clinical management were to be done in accordance with local clinical practice and regulations and at the discretion of the participating physician. Patients were to be treated at the recommended dose and schedule of each study drug per the Brazilian label in 28-day cycles for 4 induction and 2 post-ASCT consolidation cycles. Patients received daratumumab SC at 1,800 mg weekly in induction Cycles 1 and 2, then every 2 weeks in induction Cycles 3 and 4 and both post-ASCT consolidation cycles. Bortezomib was administered IV or SC at 1.3 mg/m² on Days 1, 4, 8, and 11 of each cycle. Thalidomide was administered orally at 100 mg daily in each cycle. Dexamethasone was administered IV or orally at 40 mg on Days 1, 2, 8, 9, 15, 16, 22, and 23 of induction Cycles 1 and 2; at 40 mg on Days 1 and 2 and 20 mg on Days 8, 9, 15, and 16 of induction Cycles 3 and 4; and at 20 mg on Days 1,2, 8, 9, 15, and 16 of both post-ASCT consolidation cycles. Adjustments to the administration route, dose, and schedule of bortezomib, thalidomide, and dexamethasone were at the discretion of the participating physician; however, adjustments to the daratumumab SC dose were not allowed during the study.

Data were collected retrospectively from the initiation of D-VTd therapy (baseline) up to the study inclusion visit using patient medical records (Online Resource 1, Supplemental Fig. 1), to allow for a comprehensive understanding of patients’ medical history/comorbidities, baseline demographic and disease characteristics, and intentions for therapy. Where applicable, additional data were collected prospectively from the study inclusion visit until 30 days post-consolidation via electronic case report forms, to provide comprehensive data on treatment modifications, clinical outcomes, and safety. Patients were withdrawn from data collection within the study if they were lost to follow-up, withdrew consent, died, or experienced progressive disease. If a patient prematurely discontinued D-VTd before the end of the consolidation phase, data were collected up to 30 days after the last study treatment. The end of the study was the last data collection time point for the last patient.

### Compliance with ethical standards

In accordance with local regulations, the study protocol and all protocol amendments were approved by the Independent Ethics Committee/Institutional Review Board at each study site (Online Resource 1, Supplemental Table 1). Prior to data collection, all patients (and/or legally acceptable representatives, where applicable) were informed of the voluntary and observational nature of the study and provided written informed consent.

### Study variables

Data collected at baseline and/or during the observational period in routine clinical practice included baseline patient and disease characteristics, dose exposure and modifications, clinical response, stem cell collection, ASCT outcomes, and safety outcomes. Treatment response outcomes were assessed by the participating physician in accordance with the International Myeloma Working Group response criteria [26, 27]. Complete definitions of response criteria are provided in Online Resource 1, Supplemental Table 2. Effectiveness was determined by the rate of patients achieving a ≥ VGPR and by overall response rate (ORR; partial response or better). Patients who were identified as achieving complete response (CR) in medical records but did not have a confirmatory bone marrow biopsy were reclassified as achieving VGPR. The number of collected and transplanted stem cells were recorded directly in electronic clinical report forms, and a successful ASCT was determined by the participating physician based upon the following definition: neutrophils > 0.5 × 10^9^/L, leukocytes > 1.0 × 10^9^/L, and platelets > 50 × 10^9^/L (without transfusion). Safety outcomes such as treatment-emergent adverse events (TEAEs) were documented retrospectively from baseline until the end of data collection. TEAE severity was assessed using the National Cancer Institute Common Terminology Criteria for Adverse Events Version 4. Safety outcomes of special interest included the incidence of neutropenia and infection TEAEs, which were identified using a set of grouped terms from the Medical Dictionary for Regulatory Activities (Online Resource 1, Supplemental Methods) and verbatim language provided in clinical report forms.

### Statistical analysis

No formal sample size estimation was conducted for this noninterventional study. This study aimed to enroll approximately 50 transplant-eligible patients with NDMM who received treatment with D-VTd in real-world clinical practice settings.

No formal statistical hypothesis testing was conducted. Categorial variables are summarized using frequency counts and percentages. Continuous variables are summarized using descriptive statistics.

Safety and ASCT analyses were performed based on the safety analysis population, which included all patients who received ≥ 1 dose of D-VTd. Clinical response analyses were conducted on the response-evaluable population, comprising all patients who received ≥ 1 dose of D-VTd and had ≥ 1 response assessment conducted by the participating physician. Frequencies and percentages of each response category were provided. These analyses were carried out for the entire study population, as well as separately for different treatment phases, namely induction, ASCT, and consolidation.

## Results

### Baseline patient and disease characteristics

As of the data cutoff date (August 8, 2023), a total of 51 patients were enrolled in the study, of whom 49 were included in the safety analysis population (Online Resource 1, Supplemental Fig. 2). A total of 6 (11.8%) patients discontinued treatment, and 10 (19.6%) participants discontinued the study (Online Resource 1, Supplemental Table 3). At baseline, the median (range) age of patients was 58 (38–73) years, 49.0% were male, 73.5% were White, 16.3% were Black, and the majority were of Latino ethnicity (95.9%; Table 1). Most patients had an Eastern Cooperative Oncology Group performance status of 0 (29/39 [74.4%]), and 6 (15.4%) and 4 (10.3%) patients had a performance status of 1 and ≥ 2, respectively.

**Table 1:** Demographics and baseline disease characteristics

	D-VTd (*N* = 49)
Age	
Median (range), years	58 (38–73)
Category, n (%)	
< 50 years	8 (16.3)
50 to < 60 years	21 (42.9)
60 to < 70 years	16 (32.7)
70 to < 75 years	4 (8.2)
≥ 75 years	0
Sex, n (%)	
Female	25 (51.0)
Male	24 (49.0)
Ethnicity, n (%)	
Latino^a^	47 (95.9)
Unknown	1 (2.0)
Not reported	1 (2.0)
Race, n (%)	
White	36 (73.5)
Black	8 (16.3)
Asian	1 (2.0)
Unknown	4 (8.2)
ECOG performance status, n (%)	
n	39
0	29 (74.4)
1	6 (15.4)
≥ 2	4 (10.3)
Time since initial MM diagnosis	
n	45
Median (range), months	0.7 (0.0–48.2)
Type of myeloma, n (%)	
n	43
IgA	15 (34.9)
IgG	13 (30.2)
IgM	1 (2.3)
IgD	1 (2.3)
Light chain	7 (16.3)
Biclonal	4 (9.3)
Nonsecretory myeloma	2 (4.7)
ISS staging at diagnosis, n (%)	
I	18 (36.7)
II	14 (28.6)
III	15 (30.6)
Missing	2 (4.1)

D-VTd, subcutaneous daratumumab plus bortezomib/thalidomide/dexamethasone; ECOG, Eastern Cooperative Oncology Group; Ig, immunoglobulin; ISS, International Staging System; MM, multiple myeloma

Percentage is calculated using the safety analysis population (*N* = 49) as the denominator, unless stated otherwise in the table

^a^Patients could be of Hispanic or Latino ethnicity

The median (range) time since initial MM diagnosis was 0.7 (0–48.2) months (Table 1). Among 43 patients for whom data were available, the most prevalent type of myeloma was immunoglobulin isotype A (15 [34.9%]). Among the 49 patients in the safety analysis population, at the time of diagnosis, 18 (36.7%) patients had International Staging System stage I disease, 14 (28.6%) had stage II disease, and 15 (30.6%) had stage III disease. Out of 48 patients, the majority completed 1 ASCT (47 [97.9%]) and expressed their intention to undergo consolidation therapy (45 [93.8%]).

### Treatment data

The median (range) follow-up time for this analysis was 11.5 (4.9–27.5) months, and the median (range) duration of treatment was 8.9 (1.0–15.7) months. Among the 49 patients included in the safety analysis population, 47 (95.9%) patients received ≥ 4 cycles of induction treatment, with a median (range) of 4.0 (1.0–6.0) induction cycles. A total of 37 (75.5%) patients entered the consolidation phase. During the consolidation phase, 33 (67.3%) patients had 2 treatment cycles, with a median (range) of 2.0 (1.0–2.0) consolidation cycles. The median relative dose intensities of daratumumab SC, bortezomib, thalidomide, and dexamethasone across all treatment phases are summarized in Table 2. Cycle delays were reported in 16 (32.7%) patients, including 7 (14.3%) patients who had cycle delays in response to adverse events. Notably, per label indications, daratumumab SC dose adjustments were not allowed during the study. However, dose adjustments for bortezomib, thalidomide, and dexamethasone were reported in 10 (20.4%), 5 (10.2%), and 12 (24.5%) patients, respectively. Dose interruptions occurred in 1 (2.0%) patient each for daratumumab SC, bortezomib, and dexamethasone, while 11 (22.4%) patients experienced dose interruptions for thalidomide.

**Table 2:** Summary of treatment duration, exposure, and modifications in the safety analysis population

	D-VTd (*N* = 49)
Overall treatment duration,^a^ months	
Median (range)	8.9 (1.0–15.7)
Induction	
n	49
Median (range)	3.8 (1.0–11.0)
ASCT	
n	44
Median (range)	2.6 (0.5–6.2)
Consolidation	
n	37
Median (range)	1.8 (0.3–2.4)
Number of treatment cycles	
Induction	
n	49
Median (range)	4 (1–6)
Consolidation	
n	37
Median (range)	2 (1–2)
Relative dose intensity^b^	
Daratumumab SC, %	
Induction	
Median (range)	100.0 (75.0–158.3)
Consolidation	
Median (range)	100.0 (50.0–200.0)
Bortezomib, %	
Induction	
Median (range)	99.7 (72.9–187.8)
Consolidation	
Median (range)	97.6 (41.8–114.2)
Thalidomide, %	
Induction	
Median (range)	100.0 (21.7–200.0)
Consolidation	
Median (range)	100.0 (32.1–130.4)
Dexamethasone, %	
Induction	
Median (range)	66.7 (31.3–133.3)
Consolidation	
Median (range)	66.7 (33.3–183.3)
Patients with cycle delays, n (%)	16 (32.7)
Reason for cycle delays	
Adverse event	7 (14.3)
Other	11 (22.4)
Patients with dose adjustments	
Bortezomib, n (%)	10 (20.4)
Reasons	
Adverse event	4 (8.2)
Investigator decision	6 (12.2)
Thalidomide, n (%)	5 (10.2)
Reasons	
Adverse event	5 (10.2)
Investigator decision	1 (2.0)
Dexamethasone, n (%)	12 (24.5)
Reasons	
Adverse event	0
Investigator decision	12 (24.5)
Patients with dose interruptions^c^	
Daratumumab SC	1 (2.0)
Bortezomib	1 (2.0)
Thalidomide	11 (22.4)
Dexamethasone	1 (2.0)
Patients with stopped doses^d^	
Daratumumab SC	1 (2.0)
Bortezomib	1 (2.0)
Thalidomide	9 (18.4)
Dexamethasone	1 (2.0)

ASCT, autologous stem cell transplant; D-VTd, subcutaneous daratumumab plus bortezomib/thalidomide/dexamethasone; SC, subcutaneous

^a^Overall meaning across all treatment phases (induction/ASCT/consolidation)

^b^Relative dose intensity (%) was defined as the ratio of total dose received and total dose planned

^c^Dose interruptions refer to temporary pauses in the administration of a medication, determined by the investigator, usually due to specific clinical circumstances or events

^d^Stopped doses refer to the permanent discontinuation of medication administration, as determined and implemented by the investigator

Incidence is based on number of patients, not the number of events. Therefore, 1 patient can contribute to multiple reasons for a treatment modification on different drug administration days

### Treatment response and ASCT outcomes

**Fig. 1:**
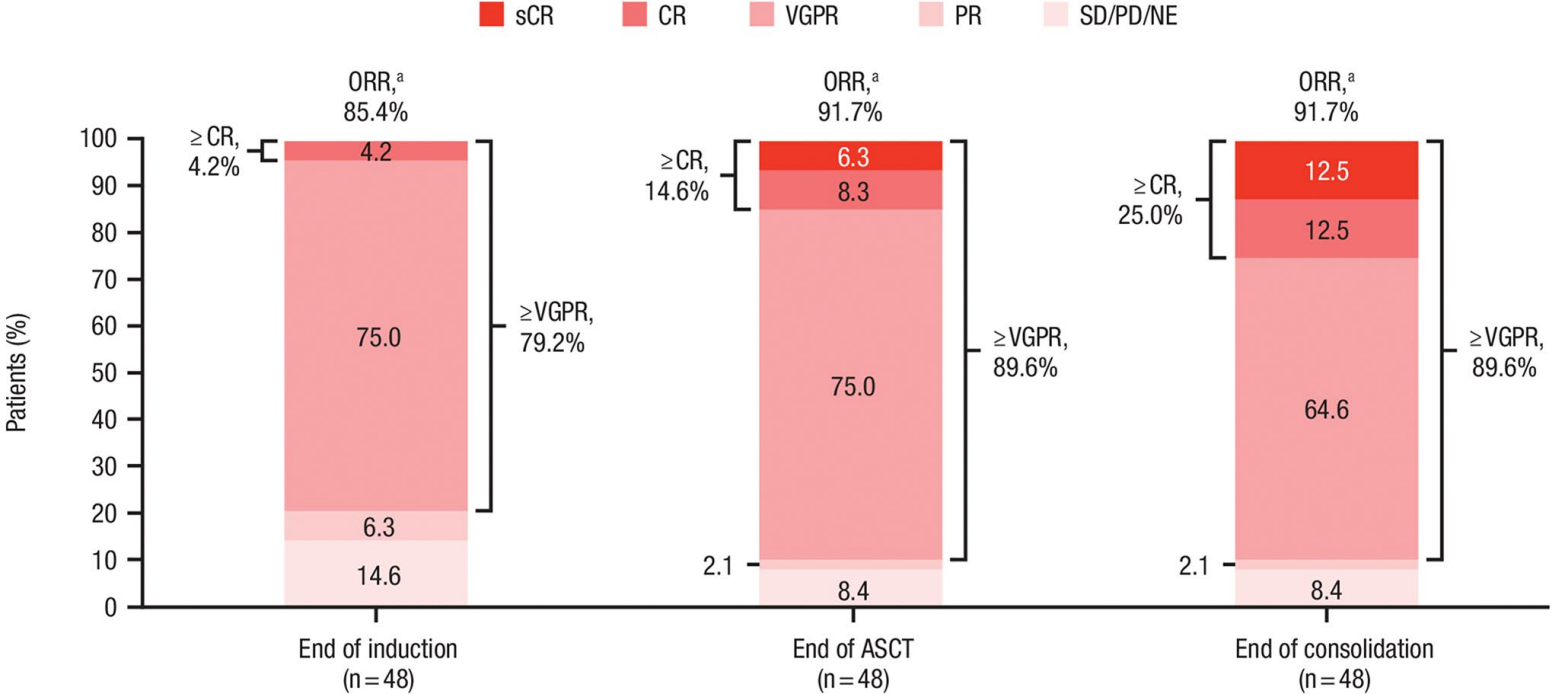
Response rates categorized by the end of each D-VTd treatment phase in the response-evaluable population. ASCT, autologous stem cell transplant; CR, complete response; D-VTd, subcutaneous daratumumab plus bortezomib/thalidomide/dexamethasone; NE, not evaluable; ORR, overall response rate; PD, progressive disease; PR, partial response; sCR, stringent complete response; SD, stable disease; VGPR, very good partial response.^a^ORR includes patients who achieved a PR or better. Rates shown are the number of patients with each type of response divided by the response-evaluable population

A summary of best response by the end of each treatment phase (induction/ASCT/consolidation) is depicted in Fig. 1. Among the 48 patients included in the response-evaluable population, 44 (91.7%) patients achieved an ORR and 43 (89.6%) achieved ≥ VGPR by the end of consolidation.

Among the 49 patients included in the safety analysis population, 45 (91.8%) patients underwent stem cell mobilization, including 38 (77.6%) patients who received granulocyte colony-stimulating factor and 20 (40.8%) patients who received plerixafor; however, it should be noted that data on the mobilization regimen used were not complete for all patients. Of the 4 patients who did not undergo stem cell mobilization, 2 had progressive disease, 1 refused ASCT, and 1 had a major protocol deviation (switched from thalidomide to lenalidomide therapy). Peripheral blood stem cell apheresis was successfully performed for 45 (91.8%) patients. Of the 37 patients for whom data were available, the median (range) number of CD34+ stem cells harvested was 5.66 (2.3–15.0)×10^6^/kg. Overall, 44 (89.8%) patients underwent ASCT (1 refused following peripheral blood stem cell apheresis), with a median (range) of 4.4 (2.3–11)×10^6^/kg CD34+ stem cells transplanted. Among the 44 patients who underwent transplantation, 43 (97.7%) patients successfully completed ASCT. Unfortunately, 1 patient died due to post-ASCT septic shock.

### Safety and tolerability

Overall, among the 49 patients included in the safety analysis population, 44 (89.8%) patients experienced ≥ 1 TEAE of any grade (Table 3), among which neutropenia (26/49 [53.1%]), peripheral neuropathy (20/49 [40.8%]), febrile neutropenia (14/49 [28.6%]), and COVID-19 (11/49 [22.4%]) were the most frequently reported. Grade 3/4 TEAEs were reported in 24 (49.0%) patients, in which febrile neutropenia (9/49 [18.4%]) and neutropenia (8/49 [16.3%]) were the most common. During the induction and consolidation phase, 11 (22.4%) of 49 and 5 (13.5%) of 37 patients reported grade 3/4 TEAEs, respectively. Serious TEAEs were reported in 14 (28.6%) patients, the majority of which occurred during the induction phase of treatment (10/49 [20.4%]); of the 14 serious TEAEs, 5 were considered to be treatment related (Online Resource 1, Supplemental Table 4). Serious TEAEs reported by ≥ 2 patients overall included COVID-19 (5/49 [10.2%]), pneumonia (2/49 [4.1%]), and febrile neutropenia (2/49 [4.1%]).

**Table 3:** Summary of TEAEs in the safety analysis population

	D-VTd (*N* = 49)
Any grade TEAE, n (%)	44 (89.8)
Treatment-related any grade TEAE^a^	35 (71.4)
Daratumumab SC	26 (53.1)
Bortezomib	29 (59.2)
Thalidomide	25 (51.0)
Dexamethasone	8 (16.3)
Most common any grade TEAEs,^b^ n (%)	
Neutropenia	26 (53.1)
Febrile neutropenia	14 (28.6)
COVID-19 infection	11 (22.4)
Peripheral neuropathy^c^	20 (40.8)
Constipation	6 (12.2)
Sinusitis	5 (10.2)
Upper respiratory tract infection	5 (10.2)
Grade 3/4 TEAE, n (%)	24 (49.0)
Treatment-related grade 3/4 TEAE	9 (18.4)
Serious TEAE, n (%)	14 (28.6)
Treatment-related serious TEAE	5 (10.2)
Daratumumab SC	4 (8.2)
Bortezomib	2 (4.1)
Thalidomide	1 (2.0)
Dexamethasone	1 (2.0)
TEAE resulting in study discontinuation, n (%)	1 (2.0)
Death due to a TEAE, n	0

AE, adverse event; D-VTd, subcutaneous daratumumab plus bortezomib/thalidomide/dexamethasone; SC, subcutaneous; TEAE, treatment-emergent adverse event

^a^Relationship to study agent is assessed by the investigator. Includes TEAEs that were very likely, probably, or possibly related to ≥ 1 of the 4 study treatments: daratumumab SC, bortezomib, thalidomide, or dexamethasone

^b^TEAEs reported in ≥ 10% of patients

^c^Neuropathy-related AEs included peripheral neuropathy, peripheral sensory neuropathy, peripheral motor neuropathy, and paresthesia

The incidence and severity of neutropenia TEAEs (based on a grouped set of preferred terms) across all treatment phases are summarized in Table 4. Overall, neutropenia TEAEs were reported in 31 (63.3%) of 49 patients, including 18 (36.7%) patients with ≥ 1 neutropenia TEAE considered related to D-VTd. By treatment phase, neutropenia TEAEs were reported in 22 (44.9%) out of 49 and 13 (35.1%) out of 37 patients during the induction and consolidation phases, respectively. Grade 3/4 neutropenia TEAEs were reported in 16 (32.7%) patients overall. By treatment phase, grade 3/4 neutropenia TEAEs were reported in 4 (8.2%) out of 49 and 3 (8.1%) out of 37 patients during the induction and consolidation phases, respectively. Overall, serious neutropenia TEAEs were reported in 2 (4.1%) patients; both of these events were classified as febrile neutropenia and unrelated to study treatment.

**Table 4:** Summary of treatment-emergent neutropenia and infections across all treatment phases in the safety analysis population

	Induction/ASCT/ consolidation (*N* = 49)	Induction (*N* = 49)	ASCT (*N* = 45)	Consolidation (*N* = 37)
Any TEAE of neutropenia,^a^ n (%)	31 (63.3)	22 (44.9)	-	13 (35.1)
≥ 1 related^b^	18 (36.7)	13 (26.5)	-	9 (24.3)
Maximum toxicity grade, n (%)				
Grade 1	7 (14.3)	7 (14.3)	-	5 (13.5)
Grade 2	6 (12.2)	8 (16.3)	-	5 (13.5)
Grade 3	14 (28.6)	4 (8.2)	-	3 (8.1)
Grade 4	2 (4.1)	0	-	0
Any TEAE of infection,^c^ n (%)	29 (59.2)	20 (40.8)	4 (8.9)	8 (21.6)
≥ 1 related^b^	7 (14.3)	4 (8.2)	0	3 (8.1)
Maximum toxicity grade, n (%)				
Grade 1	7 (14.3)	4 (8.2)	1 (2.2)	3 (8.1)
Grade 2	12 (24.5)	9 (18.4)	2 (4.4)	3 (8.1)
Grade 3	7 (14.3)	4 (8.2)	1 (2.2)	2 (5.4)
Grade 4	2 (4.1)	2 (4.1)	0	0

ASCT, autologous stem cell transplant; MedDRA, Medical Dictionary for Regulatory Activities; TEAE, treatment-emergent adverse event

^a^Treatment-emergent “neutropenia” events were identified using a set of group terms from MedDRA (see Online Resource 1, Supplemental Methods)

^b^Relationship to study agent is assessed by the investigator. Includes TEAEs that were very likely, probably, or possibly related to ≥ 1 of the 4 study treatments: daratumumab subcutaneous, bortezomib, thalidomide, or dexamethasone

^c^Treatment-emergent “infections” were identified using a set of group terms from MedDRA (see Online Resource 1, Supplemental Methods)

The incidence and severity of infection TEAEs across all treatment phases are summarized in Table 4. Overall, infection TEAEs were reported in 29 (59.2%) of 49 patients, including 7 (14.3%) patients with ≥ 1 infection TEAE considered related to D-VTd. By treatment phase, infection TEAEs were reported in 20 (40.8%) out of 49 and 8 (21.6%) out of 37 patients during the induction and consolidation phases, respectively. Grade 3/4 infection TEAEs were reported in 9 (18.4%) patients overall, the most common being COVID-19 (4/49 [8.2%]) and pneumonia (2/49 [4.1%]). The majority of grade 3/4 infection TEAEs were reported during the induction phase (6/49 [12.2%]), with 2 out of 37 (5.4%) reported in the consolidation phase. Serious infection TEAEs were reported in 10 (20.4%) patients overall, 2 (4.1%) of which were considered related to D-VTd. The most frequent (≥ 2 patients) serious infections were COVID-19 (5/49 [10.2%]) and pneumonia (2/49 [4.1%]).

Three patients (6.1%) reported injection-site reactions, which all occurred during the induction phase, were of grade 1 to 2 severity, and were considered to be related to daratumumab SC by the investigators. As of the cutoff date, 1 (2.0%) patient discontinued thalidomide study treatment due to a grade 1 TEAE of lower limb paresthesia, and 1 (2.0%) patient died due to post-ASCT septic shock that occurred 32 days after the last dose of D-VTd; this event was not considered to be related to study treatment.

## Discussion

Results from this noninterventional, multicenter, observational study provide evidence for the clinical efficacy and safety of daratumumab SC in combination with VTd in routine clinical practice for treatment-naïve, transplant-eligible patients with NDMM. Patients receiving D-VTd achieved deep responses, with 91.7% and 89.6% of patients achieving an ORR and ≥ VGPR, respectively, by the end of consolidation. Notably, these response rates are consistent with those observed in previous studies using daratumumab IV plus VTd. Furthermore, results demonstrated a safety profile consistent with those established in the previous studies using daratumumab IV in combination with VTd, with no new safety signals identified.

The clinical efficacy of D-VTd, as demonstrated in this study, is comparable to that observed in other clinical studies. Here, 89.6% of patients receiving D-VTd achieved a ≥ VGPR by the end of consolidation. This response rate is comparable to those observed in the phase 3 CASSIOPEIA studies, in which 83% of patients with NDMM receiving daratumumab IV plus VTd achieved a ≥ VGPR after consolidation [25]. In contrast, the observed rate of CR or better was slightly lower in the current study versus CASSIOPEIA (25.0% vs. 39.0%, respectively). In the current real-world study, patients with a CR had to meet all International Myeloma Working Group criteria with a bone marrow biopsy; patients considered by the attending physician to have achieved a CR without a confirmatory bone marrow biopsy were reclassified as having achieved a VGPR, and these criteria may have contributed to the observed difference between studies. Although the current study is among the first to investigate the efficacy of D-VTd with the SC formulation of daratumumab in routine clinical practice, similar results were observed in the phase 2 PLEIADES study, in which daratumumab SC was explored in combination with VRd [23]. After a median follow-up of 3.9 months, ORR was comparable to that seen in the current study (D-VRd, 97.0%; D-VTd, 91.7%) [23]. These data are further corroborated by recently published results from the PERSEUS study (median follow-up, 47.5 months), in which D-VRd resulted in an ORR of 96.6% [16]; notably, the current study was completed prior to the availability of results from the PERSEUS study. Overall, the effectiveness of daratumumab SC in combination with VTd has been demonstrated to be consistent with the established effectiveness of daratumumab IV plus VTd and other standard-of-care regimens, despite some differences in CR rates likely due to stricter criteria in the current patient cohort.

Evidence suggests that patients who undergo successful ASCT have greater subsequent survival benefits [28], thus highlighting the importance of ASCT. In the current study, 97.7% of patients who underwent ASCT reported a successful transplant, comparable to success rates reported in CASSIOPEIA and PERSEUS following daratumumab IV plus VTd and daratumumab SC plus VRd, respectively [25, 16]. Of note, the proportion of patients receiving plerixafor in the current study was higher than that reported in CASSIOPEIA (40.8% vs. 22%, respectively), and the resulting stem cell yield was lower (5.7 × 10^6^/kg vs. 6.3 × 10^6^/kg) but did not lead to ASCT failure.

The development of the SC formulation of daratumumab aims to reduce patient and provider burden by improving administration characteristics, such as a reduction in injection-site reactions. In the current study, only 3 patients (6.1%) reported injection-site reactions with daratumumab SC administration, all of which were mild (grade 1–2). This low rate is consistent with other real-world studies. In an analysis of Mayo Clinic electronic health records, of 215 patients with MM who were treated with daratumumab SC, injection-site reactions were reported in < 2% following doses 1 to 3 and < 3% cumulatively for doses 4 and onward [19]. An additional retrospective analysis of medical records from patients with MM similarly reported a low rate (9%) of injection-site reactions following daratumumab SC, with most reactions being grade ≤ 2 in severity [29]. These rates are notably lower than those reported in studies evaluating daratumumab IV, such as CASSOPEIA (35%) [25], GRIFFIN (49%) [30], and ALCYONE (27.7%) [31]. These findings suggest IV and SC formulations of daratumumab can be used somewhat interchangeably in treatment regimens, with SC administration likely being preferable due to its convenience and the associated reduction in injection-site reactions.

The safety profile of D-VTd induction/consolidation, as observed in the current study, is comparable to the safety profile for daratumumab IV plus VTd, with no new safety concerns. Among the adverse events of interest examined in the current study, grade 3/4 neutropenia and infection TEAEs were reported in 32.7% and 18.4% of patients receiving D-VTd, respectively. These frequencies are similar to those reported in CASSIOPEIA following induction/consolidation with daratumumab IV plus VTd (28% and 22%) [25]. In contrast, fewer serious TEAEs were reported in the current study compared to the number observed in CASSIOPEIA (26.8% vs. 47%); however, the number of patients discontinuing study treatment due to TEAEs during induction/consolidation remained comparably low (2% vs. 7%) [25]. Overall, daratumumab SC in combination with VTd was generally well tolerated, with clinically manageable side effects that were consistent with previous reports.

This study has some limitations, including its observational design, which can introduce selection and information bias. To minimize these biases, however, all consecutive eligible patients were enrolled, and investigators worked closely with participating centers to implement effective measures for follow-up and to capture key variables. The study included a relatively small sample size (*N* = 49), which limits the ability to make strong conclusions or evaluate outcomes in patient subgroups. Additionally, it is important to note that the study was conducted exclusively in centers across Brazil, which may limit the generalizability of data. However, Brazil remains one of the largest health care sections in Latin America, with a diverse ethnic and racial population. The current study enrolled primarily Latino patients (95.9%), with 16.3% of Black race, providing valuable global insights into the real-world efficacy and safety of D-VTd in these ethnic and racial populations.

In conclusion, daratumumab SC plus VTd demonstrated favorable responses and a safety profile that were comparable to those of its IV counterpart. Furthermore, daratumumab SC resulted in fewer injection-site reactions than were reported in prior daratumumab IV-based clinical studies, highlighting the positive impact of SC administration on patient burden. Overall, these findings gathered from a real-world clinical practice setting provide additional evidence to support the use of daratumumab SC plus VTd in transplant-eligible patients with NDMM.

## Electronic supplementary material

Below is the link to the electronic supplementary material.

Supplementary Material 1

## Data Availability

The data sharing policy of Johnson & Johnson is available at https://innovativemedicine.jnj.com/our-innovation/clinical-trials/transparency. As noted on this site, requests for access to the study data can be submitted through Yale Open Data Access (YODA) Project site at https://yoda.yale.edu
