# Supplementary material for: Real-world data on the use of subcutaneous daratumumab plus bortezomib, thalidomide, and dexamethasone in transplant-eligible patients with newly diagnosed multiple myeloma
Source: Ann Hematol. 2025 Apr 30;104(5):2787–98. doi: 10.1007/s00277-025-06365-3 (PMC12141371; doi:10.1007/s00277-025-06365-3)
Supplement: Supplementary file 1 — Supplementary Material 1 [file 277_2025_6365_MOESM1_ESM.docx]

# Online Resource 1

Real-world Data on the Use of Subcutaneous Daratumumab Plus Bortezomib, Thalidomide, and Dexamethasone in Transplant-eligible Patients With Newly Diagnosed Multiple Myeloma

Vania Hungria, Fernanda Lemos Moura, Abel Costa, Eduardo Flávio Oliveira Ribeiro, Paulo Soares, Juliana Souza Lima, Lisa Aquaroni Ricci, Celso Arrais-Rodrigues, Fabio Moore Nucci, Marinus de Moraes Lima, Roberto Jose Pessoa de Magalhães Filho, Amitabha Bhaumik, Trilok Parekh, Fredrik Borgsten, Robin Carson, Damila C. Trufelli, Edvan de Queiroz Crusoe

This file contains additional information on the MMY4046 study, including details on study design, patient disposition and flow, International Myeloma Working Group response criteria, adverse events of special interest, and serious adverse events.

**Supplemental Methods**

## Adverse events of special interest

Adverse events of special interest were events of neutropenia and infections, identified using a group of specific terms from the Medical Dictionary for Regulatory Activities and verbatim language reported on clinical report forms. The Medical Dictionary for Regulatory Activities terms for treatment-emergent neutropenia and infections were as follows:

|  | System organ class | Preferred term |
| --- | --- | --- |
| Neutropenia |  |  |
|  | BLOOD AND LYMPHATIC SYSTEM DISORDERS | AGRANULOCYTOSIS |
|  | BLOOD AND LYMPHATIC SYSTEM DISORDERS | GRANULOCYTOPENIA |
|  | BLOOD AND LYMPHATIC SYSTEM DISORDERS | NEUTROPENIA |
|  | BLOOD AND LYMPHATIC SYSTEM DISORDERS | FEBRILE NEUTROPENIA |
|  | INFECTIONS AND INFESTATIONS | NEUTROPENIC INFECTION |
|  | INFECTIONS AND INFESTATIONS | NEUTROPENIC SEPSIS |
|  | INVESTIGATIONS | GRANULOCYTE COUNT DECREASED |
|  | INVESTIGATIONS | NEUTROPHIL COUNT ABNORMAL |
|  | INVESTIGATIONS | NEUTROPHIL COUNT DECREASED |
| Infections |  |  |
|  | INFECTIONS AND INFESTATIONS | – |

# Supplemental Table 1. Independent Ethics Committees/Institutional Review Boards at each study site

| **Study site** | **Independent Ethics Committee/ Institutional Review Board** |
| --- | --- |
| Associacao Hospitalar Moinhos de Vento  Rua Ramiro Barcelos 910, Porto Alegre/RS,  CEP 90035-001 | CEP do Instituto de Educacao e Pesquisa da Associacao Hospitalar Moinhos de Vento |
| Fundação Doutor Amaral Carvalho  Rua Dona Silveria 150, Jau/SP, CEP 17210 080 | Comitê de Ética em Pesquisa do Hospital Amaral Carvalho |
| Instituto de Oncologia de Sorocaba  Av Comendador Pereira Inacio 950 Terreo, Sorocaba/SP, CEP 18030 005 | Comitê de Ética em Pesquisa da Faculdade de Ciências Médicas e da saúde PUCSP |
| Instituto D’Or de Pesquisa e Ensino IDOR  Avenida República do Líbano, 611, Ibirapuera, São Paulo/SP, CEP 04502-001 | Comitê de Ética em Pesquisa do Hospital e Maternidade São Luiz Itaim |
| Instituto D’Or de Pesquisa e Ensino IDOR  SHLS, 716, Asa Sul, Brasilia/DF, CEP 70390-700 | Comitê de Ética em Pesquisa do Hospital e Maternidade São Luiz Itaim |
| Instituto D’Or de Pesquisa e Ensino IDOR  Rua Diniz Cordeiro 30, Botafogo, Rio de Janeiro/RJ, CEP 22281 100 | Comitê de Ética em Pesquisa IDOR |
| Hospital Santa Lúcia S.A.  SHLS, Quadra 716 - Conjunto C - Blocos A - B  e C, Brasília/DF, CEP 70390-700 | Comitê de Ética Invitare (closed activities at the end of the study) |
|  | Comitê de Ética do Hospital Alemão Oswaldo Cruz |
| Hospital São Rafael  Av. São Rafael, 2152 - São Marcos, Salvador/BA, CEP 41253-190 | Comitê de Ética em Pesquisa - Monte Tabor - Hospital São Rafael |
| Hospital Israelita Albert Einstein  Avenida Albert Einstein, 627, São Paulo/SP,  CEP 05651-901 | Comitê de Ética em Pesquisa do Hospital Israelita Albert Einstein |
| Clinica São Germano  Comendador Miguel Calfat 217, São Paulo/SP, CEP 01455 010 | CONEP - Comissao Nacional de Etica em Pesquisa |
|  | Comitê de Ética Invitare (closed activities at the end of the study) |
|  | Comitê de Ética do Hospital Alemão Oswaldo Cruz |
| Instituto de Educacao Pesquisa e Gestao em Saude Instituto Americas (COI)  Avenida Jorge Curi, 550, Barra da Tijuca, Rio de Janeiro/RJ, CEP 22793-080 | Comitê de Ética em Pesquisa em Seres Humanos do Hospital Pró-Cardíaco (CEP/HPC) |
| Fundação Antonio Prudente – AC Camargo Cancer Center  Rua Prof Antonio Prudente, 211, Liberdade,  São Paulo/SP, CEP 01509 900 | Comitê de Ética em Pesquisa em Seres Humanos Fundação Antônio Prudente - Hospital AC Camargo |
| Sociedade Beneficente de Senhoras - Hospital Sirio Libanes HSL Unidade Brasilia  SGAS 614, Conjunto C – Salas 17 a 24 – Subsolo, Brasilia/DF, CEP 07020-730 | Comitê de Ética em Pesquisa da Sociedade Beneficente de Senhoras Hospital Sirio-Libanes (CEPesq/HSL) |
| Sociedade Beneficente de Senhoras Hospital Sirio Libanes  Rua Dona Adma Jafet, 91, São Paulo/SP, CEP 01308 901 | Comitê de Ética em Pesquisa da Sociedade Beneficente de Senhoras Hospital Sirio-Libanes (CEPesq/HSL) |
| Instituto D’Or de Pesquisa e Ensino IDOR  Rua das Fronteiras, 175, Boa Vista, Recife/PE, CEP 50070-170 | Comitê de Ética em Pesquisa Envolvendo Seres Humanos do Instituto de Medicina Integral Prof. Fernando Figueira CEP-IMIP |
| Impar Servicos Hospitalares S/A  Rua La Sale, 12, Centro - Niteroi, Rio de Janeiro/RJ, CEP 24020-096 | Comitê de Ética em Pesquisa da Esho Empresa de Serviços Hospitalares – Hospital Pró-cardiáco |
| Hospital Nove de Julho  Rua Peixoto Gomide, 263, São Paulo/SP, CEP 01409-902 | Comitê de Ética em Pesquisa do Hospital 9 de Julho |
| Instituto Hematologia e Oncologia Curitiba - Clinica Medica S.A.  Rua Fagundes Varela, 1785, Curitiba/PR, CEP 82520-040 | Comitê de Ética em Pesquisa em Seres Humanos do HC/UFPR |

# Supplemental Table 2. IMWG response criteria definitions [1, 2]

| **Response** | **Response criteria** |
| --- | --- |
| sCR | - CR as defined below, *and* - Normal FLC ratio, *and* - Absence of clonal PCs by immunohistochemistry, immunofluorescence,^a^ or 2- to 4-color flow cytometry |
| CR^b^ | - Nonsecretory myeloma in the serum and urine, *and* - Disappearance of any soft tissue plasmacytomas, *and* - <5% PCs in bone marrow |
| VGPR^b^ | - Serum and urine M-component detectable by immunofixation but not on electrophoresis, *or* - ≥90% reduction in serum M-protein plus urine M-protein <100 mg/24 hours |
| PR | - ≥50% reduction of serum M-protein and reduction in 24-hour urinary M-protein by ≥90% or to <200 mg/24 hours, *or* - If the serum and urine M-protein are not measurable, a decrease of ≥50% in the difference between involved and uninvolved FLC levels is required, *or* - If serum and urine M-protein are not measurable, and serum free light assay is also not measurable, ≥50% reduction in bone marrow PCs is required, provided baseline bone marrow plasma cell percentage is ≥30%, *and* - In addition to the above criteria, if present at baseline, a ≥50% reduction in the size of soft tissue plasmacytomas is also required |
| Stable disease | - Not meeting criteria for CR, VGPR, PR, or PD |
| PD^c^ | Increase of 25% from lowest response value in any one of the following:   - Serum M-component (absolute increase must be ≥0.5 g/dL) - Urine M-component (absolute increase must be ≥200 mg/24 hours) - Only in patients without measurable serum and urine M-protein levels: the difference between involved and uninvolved FLC levels (absolute increase must be >10 mg/dL) - Only in patients without measurable serum and urine M-protein levels and without measurable disease by FLC levels: bone marrow PC percentage (absolute percentage must be ≥10%) - Bone marrow PC percentage: absolute percentage must be >10% - Definite development of new bone lesions or soft tissue plasmacytomas or definite increase in the size of existing bone lesions or soft tissue plasmacytomas - Development of hypercalcemia (corrected serum calcium >11.5 mg/dL) that can be attributed solely to the PC proliferative disorder |

CR, complete response; FLC, free light chain; IMWG, International Myeloma Working Group; PC, plasma cell; PD, progressive disease; PR, partial response; sCR, stringent complete response; SD, stable disease; VGPR, very good partial response.

^a^Presence/absence of clonal cells is based upon the kappa/lambda ratio. An abnormal kappa/lambda ratio by immunohistochemistry or immunofluorescence requires a minimum of 100 PCs for analysis. An abnormal ratio reflecting presence of an abnormal clone is kappa/lambda of >4:1 or <1:2.

^b^Clarifications to IMWG criteria for coding CR and VGPR in patients in whom the only measurable disease is by serum FLC levels: CR in such patients indicates a normal FLC ratio of 0.26 to 1.65 in addition to CR criteria listed above. VGPR in such patients requires a >90% decrease in the difference between involved and uninvolved FLC levels.

^c^Clarifications to IMWG criteria for coding PD: Bone marrow criteria for PD are to be used only in patients without measurable disease by M-protein and by FLC levels; “25% increase” refers to M-protein, FLC, and bone marrow results, and does not refer to bone lesions, soft tissue plasmacytomas, or hypercalcemia. The “lowest response value” does not need to be a confirmed value.

All response categories (CR, sCR, VGPR, PR, and PD) require 2 consecutive assessments made at any time before the institution of any new therapy; CR, sCR, VGPR, PR, and SD categories also require no known evidence of progressive or new bone lesions if radiographic studies were performed. VGPR and CR categories require serum and urine studies regardless of whether disease at baseline was measurable on serum, urine, both, or neither. Radiographic studies are not required to satisfy these response requirements. Bone marrow assessments need not be confirmed. For PD, serum M-component increases of ≥1 g/dL are sufficient to define relapse if starting M-component is ≥5 g/dL.

# Supplemental Table 3. Summary of patient disposition

| **n (%)** | **D-VTd** |
| --- | --- |
| Enrolled | 51 (100) |
| Patients enrolled but not treated per protocol^a^ | 2 (3.9) |
| Patients who discontinued treatment | 6 (11.8) |
| Reason for discontinuation |  |
| Other | 4 (7.8) |
| Physician recommendation | 1 (2.0) |
| Study terminated by sponsor | 1 (2.0) |
| Patients who discontinued study | 10 (19.6) |
| Reason for discontinuation |  |
| Other | 6 (11.8) |
| Progressive disease | 2 (3.9) |
| Death | 1 (2.0) |
| Study terminated by sponsor | 1 (2.0) |

D-VTd, subcutaneous daratumumab plus bortezomib/thalidomide/dexamethasone.

^a^Two patients were treated outside the institution.

# Supplemental Table 4. Treatment-related serious TEAEs in the safety analysis population

|  |  | **D-VTd**  **n (%)** | | | |
| --- | --- | --- | --- | --- | --- |
|  | **Total** | **Related to daratumumab** | **Related to bortezomib** | **Related to thalidomide** | **Related to dexamethasone** |
| Analysis set: safety | 49 | – | – | – | – |
| Total number of patients with serious TEAEs related to study treatment, n (%) | 5 (10.2) | 4 (8.2) | 2 (4.1) | 1 (2.0) | 1 (2.0) |
| MedDRA system organ class/preferred term, n (%) |  |  |  |  |  |
| Infections and infestations | 2 (4.1) | 2 (4.1) | 1 (2.0) | 0 | 0 |
| Escherichia sepsis | 1 (2.0) | 1 (2.0) | 0 | 0 | 0 |
| Pneumonia | 1 (2.0) | 1 (2.0) | 1 (2.0) | 0 | 0 |
| Sinusitis | 1 (2.0) | 1 (2.0) | 1 (2.0) | 0 | 0 |
| Blood and lymphatic system disorders | 1 (2.0) | 1 (2.0) | 1 (2.0) | 0 | 1 (2.0) |
| Anemia | 1 (2.0) | 1 (2.0) | 1 (2.0) | 0 | 1 (2.0) |
| Gastrointestinal disorders | 1 (2.0) | 0 | 0 | 1 (2.0) | 0 |
| Constipation | 1 (2.0) | 0 | 0 | 1 (2.0) | 0 |
| General disorders and administration site conditions | 1 (2.0) | 1 (2.0) | 0 | 0 | 0 |
| Pyrexia | 1 (2.0) | 1 (2.0) | 0 | 0 | 0 |
| Renal and urinary disorders | 1 (2.0) | 1 (2.0) | 1 (2.0) | 0 | 1 (2.0) |
| Acute kidney injury | 1 (2.0) | 1 (2.0) | 1 (2.0) | 0 | 1 (2.0) |

D-VTd, subcutaneous daratumumab plus bortezomib/thalidomide/dexamethasone; MedDRA, Medical Dictionary for Regulatory Activities; TEAE, treatment-emergent adverse event.

Patients are counted only once for any given event, regardless of the number of times they actually experienced the event. TEAEs are coded using MedDRA version 26.0.

**Supplemental Fig. 1** Study design

**
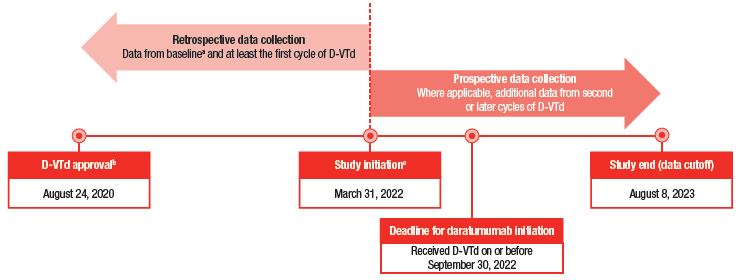
**

D-VTd, subcutaneous daratumumab plus bortezomib/thalidomide/dexamethasone; ICF, informed consent form.

^a^Baseline was defined as the start of D-VTd therapy.

^b^Approval in Brazil.

^c^Patients must have provided informed consent prior to study initiation.

**Supplemental Fig. 2** Patient flow diagram

**
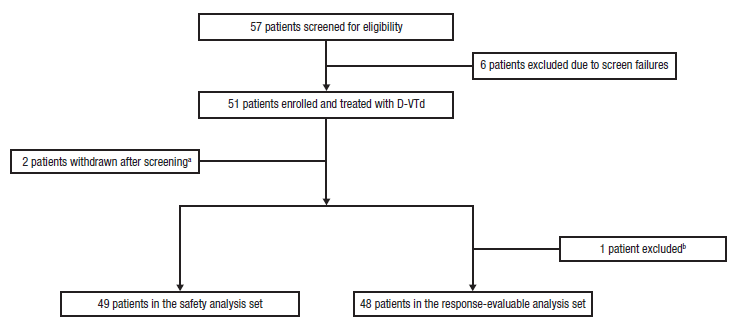
**

D-VTd, subcutaneous daratumumab plus bortezomib/thalidomide/dexamethasone.

^a^Two patients were excluded from the safety analysis population because they received treatment at a different institution.

^b^One patient was excluded due to switching treatments from thalidomide to lenalidomide before the first response assessment was performed.

**References**

1. Durie BGM, Harousseau JL, Miguel JS, Blade J, Barlogie B, Anderson K, Gertz M, Dimopoulos M, Westin J, Sonneveld P, Ludwig H, Gahrton G, Beksac M, Crowley J, Belch A, Boccadaro M, Cavo M, Turesson I, Joshua D, Vesole D, Kyle R, Alexanian R, Tricot G, Attal M, Merlini G, Powles R, Richardson P, Shimizu K, Tosi P, Morgan G, Rajkumar SV (2006) International uniform response criteria for multiple myeloma. Leukemia 20:1467-1473. https://doi.org/10.1038/sj.leu.2404284
2. Rajkumar SV, Harousseau JL, Durie B, Anderson KC, Dimopoulos M, Kyle R, Blade J, Richardson P, Orlowski R, Siegel D, Jagannath S, Facon T, vet-Loiseau H, Lonial S, Palumbo A, Zonder J, Ludwig H, Vesole D, Sezer O, Munshi NC, San MJ (2011) Consensus recommendations for the uniform reporting of clinical trials: report of the International Myeloma Workshop Consensus Panel 1. Blood 117:4691-4695. https://doi.org/10.1182/blood-2010-10-299487
